# Upregulated microRNA‐450b‐5p represses the development of acute liver failure via modulation of liver function, inflammatory response, and hepatocyte apoptosis

**DOI:** 10.1002/iid3.767

**Published:** 2023-02-24

**Authors:** Jun Fang, Jing Kuang, Shuli Hu, Xiuhong Yang, Weibo Wan, Jing Li, Xuepeng Fan

**Affiliations:** ^1^ Department of Liver‐Gallbladder and Gastric Diseases Wu Han Hospital of Traditional Chinese Medicine Wuhan Hubei People's Republic of China; ^2^ Department of Intensive Care Unit Wuhan No. 1 Hospital Wuhan Hubei People's Republic of China; ^3^ Department of Internal Medicine‐Cardiovascular Wuhan No. 1 Hospital Wuhan Hubei People's Republic of China

**Keywords:** acute liver failure, apoptosis, inflammatory response, microRNA‐450b‐5p, Mouse Double Minute 2 protein, pathological change

## Abstract

**Objective:**

It has been evidenced that microRNAs (miRs) exert crucial effects on acute liver failure (ALF), while the detailed function of miR‐450b‐5p in ALF progression remained obscure. The purpose of this research was to unravel the regulatory mechanism of miR‐450b‐5p in ALF via modulating Mouse Double Minute 2 protein (MDM2).

**Methods:**

ALF was induced in mice by intraperitoneal injection of d‐galactosamine ( d‐GalN) and lipopolysaccharide (LPS). Adenoviruses containing overexpressed miR‐450b‐5p, MDM2 shRNA, and overexpressed MDM2 sequences were utilized to manipulate miR‐450b‐5p and MDM2 expression in the liver before the mice were treated with d‐GalN/LPS‐induced ALF. Subsequently, miR‐450b‐5p and MDM2 expression levels in liver tissues of ALF mice were examined. Serum biochemical parameters of liver function were tested, serum inflammatory factors were assessed, and the histopathological changes and hepatocyte apoptosis in liver tissues were observed. The relation between miR‐450b‐5p and MDM2 was verified.

**Results:**

In ALF mice, miR‐450b‐5p was low‐expressed while MDM2 was high‐expressed. The upregulation of miR‐450b‐5p or downregulation of MDM2 could alleviate liver function, mitigate the serum inflammatory response and pathological changes in liver tissues, as well as inhibit the apoptosis of hepatocytes. MiR‐450b‐5p targeted MDM2. MDM2 overexpression reversed the repressive effects of elevated miR‐450b‐5p on ALF.

**Conclusion:**

The upregulated miR‐450b‐5p blocks the progression of ALF via targeting MDM2. This study contributes to affording novel therapeutic targets for ALF treatment.

## Graphical abstract

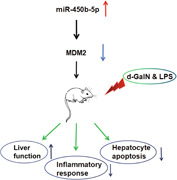

## 1 INTRODUCTION

Acute liver failure (ALF) is featured by an abrupt occurrence of liver‐based coagulopathy and hepatocellular damage that is attributed to severe degeneration in liver cell function.^1^ The major etiologies of ALF include acetaminophen overdose, viral hepatitis, drug‐caused liver impairments, Wilson's disease, and autoimmune hepatitis.^2^ There are multiple complications of ALF, such as encephalopathy, cerebral edema, sepsis, renal failure, gastrointestinal bleeding, and respiratory failure.^3^ ALF mortality has been improved with the extensive utilization of high‐volume plasma exchange and other medical therapies, yet the cornerstone of ALF management is still liver transplantation.^4^ However, organ shortage remains one of the main impediments to liver transplantation; the inaccurate selection for transplantation also poses great obstacles to ALF treatment.^5^ Therefore, novel alternative therapeutic targets for ALF require further exploration to improve such a challenging scenario.

MicroRNAs (miRNAs) have been acknowledged as pivotal modulators in most developmental and pathological processes, including those in liver development.^6^ Some hepatic miRNA signatures are even implicated in regeneration in auxiliary liver transplantation.^7^ For instance, Shao et al.^8^ have elucidated that amplified miR‐455‐3p can ameliorate macrophage infiltration and local liver injury, thereby relieving liver histology and systemic disorder in a mouse model of acute liver injury. miR‐125b‐5p mimic in mouse liver mitigates damage and improves survival in the ALF mouse model.^9^ miR‐450 has been revealed to restrict the biological behavior of HepG2 cells, offering a theoretical basis for liver cancer treatment.^10^ As for miR‐450b‐5p, it has been revealed to be a potential biomarker in liver self‐healing plasmodium malaria and transient ischemic attack.^11^, ^12^ Meanwhile, miR‐450b‐5p has been reported to be involved in hepatic ischemia/reperfusion injury,^13^ and modulate the progression of protective immunity against malarial blood stages of *Plasmodium chabaudi* in the female mouse liver,^11^ while its function in ALF remained largely unclear. It was predicted through the bioinformatics website that miR‐450b‐5p had a target gene Mouse Double Minute 2 protein (MDM2). As a cellular modulator of p53 tumor inhibitor,^14^ MDM2 has been reported to exert crucial influences in promoting liver regeneration.^15^ Furthermore, the overexpression of MDM2 in the livers of rats has been observed, and depleted expression of MDM2 contributes to attenuating hepatocarcinogenesis.^16^ While in ALF, the MDM2 function was not extensively explored. As stated above, miR‐450b‐5p and MDM2 exhibited a pivotal role in liver‐related disease, while their regulatory mechanisms in ALF development remained obscure. This study was conducted to unravel the function of miR‐450b‐5p in ALF via modulating MDM2, affording promising therapeutic candidates for ALF treatment.

## 2 MATERIAL AND METHODS

### 2.1 The establishment and grouping of the ALF mouse model

Adult male C57BL/6J mice (aged 8 w) were provided by the animal center of Wuhan University (Wuhan, China). Mice were fed in a specific pathogen‐free laboratory with 60%–65% humidity at 22–25°C for 7 d.

All mice were fasted for 12 h before modeling yet with water supply. Next, the ALF mice were intraperitoneally injected with 800 mg kg^−1^ d‐galactosamine (d‐GalN; Sigma Aldrich) and 10 µg kg^−1^ lipopolysaccharide (LPS; Sigma Aldrich) to establish the ALF mouse model.^17^ An equal volume of normal saline was injected into the normal control mice.

Eighty‐four mice were randomly classified into eight groups (*n* = 12): control group, ALF group, ALF + (Ad‐miR‐negative control [NC]) group, ALF + (Ad‐miR‐450b‐5p) group, ALF + (Ad‐short hairpin RNA [sh]‐NC) group, ALF + (Ad‐shMDM2) group, ALF + (Ad‐miR‐450b‐5p + overexpression [oe]‐NC) group, and ALF + (Ad‐miR‐450b‐5p + oe‐MDM2) group.

**Figure 1:**
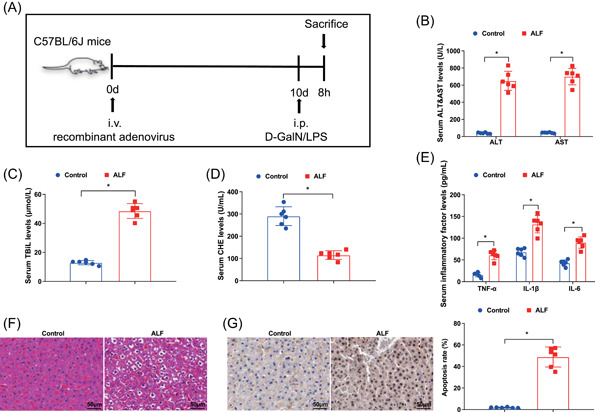
ALF mouse model is successfully established. (A) A schematic illustration of the experimental design; (B–D) the concentration of ALT, AST, TBiL, and CHE was detected by automatic biochemical analyzer; (E) the contents of serum inflammatory factors were examined by ELISA; (F) the pathological changes of liver tissues in ALF mice was assessed by HE staining; (G) the hepatocyte apoptosis in ALF mice was tested by TUNEL staining. *p < .05. ALF, acute liver failure; ALT, alanine aminotransferase; AST, aspartate aminotransferase; CHE, cholinesterase; ELISA, enzyme‐linked immunosorbent assay; HE, hematoxylin‐eosin; TBiL, total bilirubin; TUNEL, transferase‐mediated deoxyuridine triphosphate‐biotin nick end labeling.

Except for the control group and ALF group, mice in other groups were injected with corresponding recombine adenovirus vectors with a volume of 200 μl containing 2 × 10^9^ infective units of viruses through the tail vein at 10 days before d‐GalN/LPS treatment. The adenoviruses used in the experiment were synthesized by Shanghai GenePharma Co., Ltd. After 8 h of d‐GalN/LPS treatment, mice were euthanized by anesthesia overdose. Blood collection from the abdominal aorta was used for detecting liver function indicators and inflammatory factors. The mice's livers were removed for histological examination, western blot, and quantitative real‐time polymerase chain reaction analysis (RT‐qPCR) analysis.^18^ The schematic illustration of the experimental design is shown in Figure 1A.

### 2.2 Production of recombinant adenoviruses particles

miR‐450b‐5p, MDM2 shRNA, and MDM2 sequences were inserted into the adenovirus shuttle plasmid pDC316 to construct pDC316‐miR‐450b‐5p, pDC316‐MDM2 shRNA, and pDC316‐MDM2. The constructed recombinant shuttle plasmid and the adenovirus helper plasmid pBHGloxΔE1, 3Cre were transfected into HEK‐293 cells using Lipofectamine 3000 (Thermo Fisher Scientific) to obtain the recombinant adenovirus (Ad‐miR‐450b‐5p, Ad‐shMDM2, and Ad‐oe‐MDM2). After the recombinant adenovirus was identified correctly by PCR, it was amplified and purified. Later, the recombinant adenovirus titers were determined by TCID50.

### 2.3 Detection of serum biochemical indices

After 10 min centrifugation of the blood at 300*g* at 20°C, the serum was separated. The concentrations of alanine aminotransferase (ALT), aspartate aminotransferase (AST), total bilirubin (TBiL), and cholinesterase (CHE) were then examined using a fully automatic biochemical analyzer (Hitachi 7170; Hitachi High Technologies Corp.). These biochemical indices were detected to validate the successful construction of ALF mouse models and to assess the degree of liver injury.^18^, ^19^

### 2.4 Enzyme‐linked immunosorbent assay (ELISA)

The contents of serum tumor necrosis factor‐α (TNF‐α), interleukin (IL)‐1β, and IL‐6 were detected by ELISA kit (ADL) concerning manufacturers' instructions.

### 2.5 Hematoxylin‐eosin (HE) staining

The liver tissues were fixed with 4% paraformaldehyde for 24 h, dehydrated with gradient ethanol, penetrated with 1/2 xylene and 1/2 ethanol, and embedded with paraffin and sliced. Then, the liver tissues were subjected to 15‐min staining with hematoxylin, 10‐s differentiation with hydrochloric acid, 1‐min washing with tap water, 3‐min staining with 0.5% eosin, and finally 5‐min soaking in ethanol. After permeabilization and blocking, the morphological changes in liver tissues were observed under a microscope and the image was captured.^20^

### 2.6 Transferase‐mediated deoxyuridine triphosphate‐biotin nick end labeling (TUNEL) staining

The liver cell apoptosis (Beijing Zhongshu GoldenBridge Biotechnology Co., Ltd.) was assessed by TUNEL staining. Sections were prepared with diaminobenzidine and stained with hematoxylin. A light microscope (Olympus Optical Co., Ltd.) was adopted to observe the apoptotic cells. Apoptotic index = quantity of apoptotic cells/total quantity of cells × 100%.^20^

### 2.7 Reverse‐transcription quantitative polymerase chain reaction (RT‐qPCR)

The TRIzol reagent (Life Technologies) was used for the total RNA extraction. The extracted RNA was then diluted in proportion; and the extracted RNA concentration was measured with a cuvette on the ultrafine nucleic acid quantitative spectrometer (Thermo Nanodrop 1000; Thermo Fisher Scientific). Each sample was repeated three times, and the data was recorded. The reverse transcription was performed as the instructions of the miRNA reverse transcriptase (NCode miRNA First‐Strand cDNA Synthesis Kit; Invitrogen) and reverse transcriptase kit (RevertAid First‐Strand cDNA Synthesis Kit; Thermo Scientific). Real‐time PCR was conducted based on the instructions of the SYBR Green qPCR Master Mix (Fermentas). U6 was set as the endogenous reference of miR‐450b‐5p and MDM2 was that of glyceraldehyde phosphate dehydrogenase (GAPDH). The difference in target gene RNA expression was compared using the $2^{‐∆∆C_{t}}$ method.^20^ The RT‐qPCR primers are shown in Supporting Information: Table S1.

### 2.8 Western blot analysis

After protein extraction from the liver tissues, the protein levels were tested by a bicinchoninic acid protein content detection kit (Nanjing Keygen Biotechnology Co., Ltd.). The extracted high‐temperature denatured protein was mixed with loading buffer, boiled at 95°C for 10 min, treated with 10% sodium dodecyl sulphate polyacrylamide gel electrophoresis, and transferred to polyvinylidene fluoride (PVDF) membrane. The protein was then blocked with 5% skimmed milk powder for 2.5–3 h. Then, the PVDF membrane was subjected to incubation with diluted primary antibody against MDM2 (1:1000; Santa Cruz Biotechnology), and GAPDH (1:1000; Santa Cruz Biotechnology) at 4°C overnight, followed by 2‐h incubation with the relative secondary antibody. Then, equal amounts of A and B solutions of the enhanced chemiluminescence (ECL) kit (Ameshame) were mixed thoroughly and dropped on PVDF membrane. Image development was performed using the gel imaging system (Bio‐Rad Laboratories, Inc.). After the image was scanned and saved, the gray value of band was analyzed.

### 2.9 Dual‐luciferase reporter gene assay

The binding sites of miR‐450b‐5p to MDM2 were predicted through the bioinformatics website (https://starbase.sysu.edu.cn). The binding sequences and its corresponding mutation sequences were inserted into the pmirGLO vector (Promega) to construct pmirGLO‐MDM2‐Wt (wild type) and pmirGLO‐MDM2‐Mut (mutation type).

Mouse hepatocyte AML12 cells (Procell) were seeded onto 12‐well plates and then cotransfected with 500 ng pmirGLO‐MDM2‐Wt and pmirGLO‐MDM2‐Mut and 100 nM miR‐450b‐5p mimic or it NC with Lipofectamine 3000 transfection reagent. Subsequently, cell lysates were harvested 48 h posttransfection, followed by quantification of firefly and *Renilla* luciferase activities with the Dual‐Luciferase Reporter Assay System (Promega) on a GLOMAX 20/20 luminometer (Promega). Relative activity was counted with the ratio of firefly luciferase activity to *Renilla* luciferase activity.

### 2.10 Statistical analysis

All data were statistically analyzed using SPSS 22.0 software (IBM Corp.) and GraphPad Prism 8.02 (GraphPad Software). All the experimental data were represented as mean ± standard deviation. Comparisons between two groups were made by Student's unpaired *t* test. *p* < .05 was regarded as an indicator of statistical significance.

## 3 RESULTS

### 3.1 ALF mouse model is successfully established

To unravel the pathogenesis of ALF, we first used d‐GalN and LPS to induce a typical model of ALF in mice. As suggested by serum biochemical parameters, the concentrations of serum ALT and AST were elevated in ALF mice; TBiL was also increased while CHE level was depleted in ALF mice (Figure 1B–D). ELISA was adopted to examine the inflammatory factor levels in the serum of experimental mice. It was uncovered that ALF mice also displayed high levels of TNF‐α, IL‐1β, and IL‐6 (Figure 1E).

The results of HE staining showed that the liver tissues of ALF mice exhibited obvious hepatic plate disorder, and the congestion of hepatic sinusoids was saliently enlarged after HE staining. Numerous cells displayed marked degeneration, necrosis, karyopyknosis, chromatin condensation, nuclear fragmentation or lysis, and numerous red blood cells in peripheral liver tissue were infected (Figure 1F).

Apoptosis was detected by TUNEL staining, which indicated that TUNEL‐positive cells were observed in the liver tissues of normal rats, while the number of TUNEL‐positive cells in ALF mice was amplified (Figure 1G).

These findings reflected that the ALF mouse model was established successfully.

### 3.2 miR‐450b‐5p elevation alleviates liver function, decreases the levels of inflammatory factors in serum, and inhibits hepatocyte apoptosis

**Figure 2:**
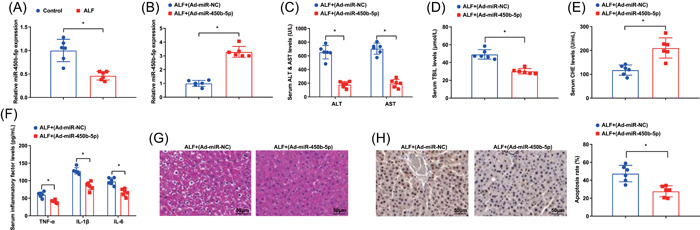
miR‐450b‐5p elevation alleviates liver function, decreases the contents of inflammatory factors in serum, and inhibits hepatocyte apoptosis. (A) miR‐450b‐5p expression in the liver tissues of ALF mice was examined by RT‐qPCR; (B) miR‐450b‐5p expression in the liver tissues of ALF mice after miR‐450b‐5p upregulation was tested by RT‐qPCR; (C–E) the concentration of ALT, AST, TBiL, and CHE in ALF mice after miR‐450b‐5p upregulation was detected by automatic biochemical analyzer; (F) the contents of serum inflammatory factors in ALF mice after miR‐450b‐5p upregulation were examined by ELISA; G, the pathological changes of liver tissues in ALF mice after miR‐450b‐5p upregulation was assessed by HE staining; (H) the hepatocyte apoptosis in ALF mice after miR‐450b‐5p upregulation was tested by TUNEL staining. *p < .05. ALF, acute liver failure; ALT, alanine aminotransferase; AST, aspartate aminotransferase; CHE, cholinesterase; ELISA, enzyme‐linked immunosorbent assay; HE, hematoxylin‐eosin; RT‐qPCR, reverse‐transcription quantitative polymerase chain reaction; TBiL, total bilirubin; TUNEL, transferase‐mediated deoxyuridine triphosphate‐biotin nick end labeling.

Subsequently, we probed the impacts of miR‐450b‐5p on the development of ALF. miR‐450b‐5p expression in the liver tissues of ALF mice was measured by RT‐qPCR, which revealed that miR‐450b‐5p was lowly expressed in the liver tissues of ALF mice (Figure 2A).

To confirm the influence of miR‐450b‐5p augmentation on ALF mice, we upregulated miR‐450b‐5p expression in ALF mice, and RT‐qPCR reflected that miR‐450b‐5p expression was amplified in ALF mice after being injected with Ad‐miR‐450b‐5p (Figure 2B). Affected by miR‐450b‐5p overexpression in ALF mice, ALT, AST, and TBiL levels were reduced, while CHE level was elevated (Figure 2C–E). Moreover, in response to the upregulated miR‐450b‐5p, the contents of TNF‐α, IL‐1β, and IL‐6 were reduced in ALF mice (Figure 2F). Furthermore, the hepatic cord was arranged radially in the liver tissues of ALF mice after upregulation of miR‐450b‐5p, with mild edema of liver cells and clear hepatic sinusoids (Figure 2G). Decelerated apoptosis was also observed in ALF mice after miR‐450b‐5p elevation (Figure 2H).

These discoveries unveiled that miR‐450b‐5p displayed a low level in liver tissues of ALF mice, and miR‐450b‐5p amplification could relieve the degree of liver injury and inflammatory response, as well as dampen hepatocyte apoptosis.

### 3.3 miR‐450b‐5p targets MDM2

**Figure 3:**
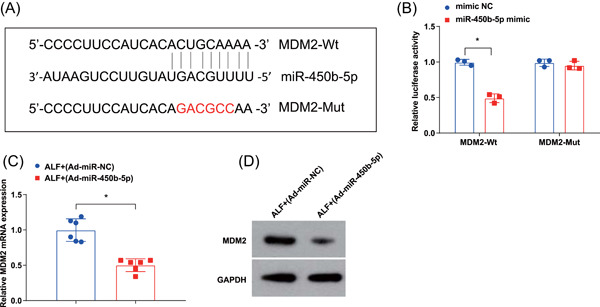
miR‐450b‐5p targets MDM2. (A) the binding sites between miR‐450b‐5p and MDM2 were predicted by bioinformatics website; (B) the targeting relation between miR‐450b‐5p and MDM2 was validated by dual luciferase reporter gene assay; (C, D) MDM2 expression after the upregulation of miR‐450b‐5p was detected by RT‐qPCR and western blot analysis. *p < .05. MDM2, Mouse Double Minute 2 protein; RT‐qPCR, reverse‐transcription quantitative polymerase chain reaction.

The bioinformatics website (https://starbase.sysu.edu.cn/) predicted that miR‐450b‐5p could bind to MDM2 (Figure 3A). As suggested by dual luciferase reporter gene assay, miR‐450b‐5p impaired the luciferase activity of pmirGLO‐MDM2‐Wt (Figure 3B), indicating that MDM2 was targeted by miR‐450b‐5p. Moreover, RT‐qPCR and Western blot analysis disclosed a significant reduction in MDM2 expression in ALF mice after being treated with Ad‐miR‐450b‐5p (Figure 3C,D). These findings evidenced that miR‐450b‐5p targeted MDM2.

### 3.4 MDM2 silencing ameliorates liver function, relieves inflammatory response in serum and restrains hepatocyte apoptosis

**Figure 4:**
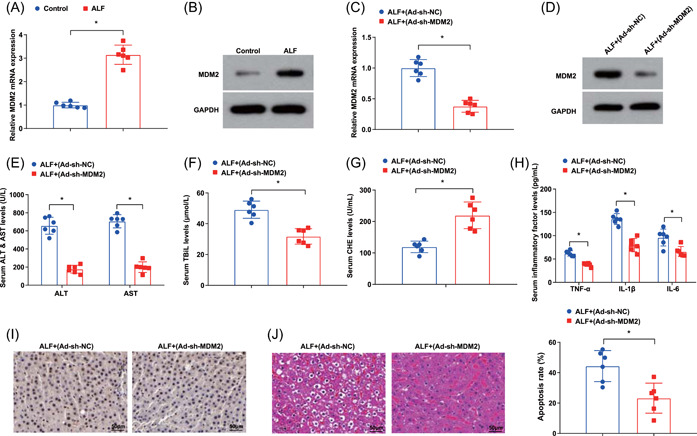
MDM2 silencing ameliorates liver function, relieves inflammatory response in serum and restrains hepatocyte apoptosis. (A, B) MDM2 expression in liver tissues of ALF mice was detected by RT‐qPCR and western blot analysis; (C, D) MDM2 expression in liver tissues of ALF mice after MDM2 silencing was detected by RT‐qPCR and western blot analysis; (E–G) the concentration of ALT, AST, TBiL and CHE in ALF mice after MDM2 silencing was detected by automatic biochemical analyzer; (H) the contents of serum inflammatory factors in ALF mice after MDM2 silencing were examined by ELISA; (I) the pathological changes of liver tissues in ALF mice after MDM2 silencing was assessed by HE staining; (J) the hepatocyte apoptosis in ALF mice after MDM2 silencing was tested by TUNEL staining. *p < .05. ALF, acute liver failure; ALT, alanine aminotransferase; AST, aspartate aminotransferase; CHE, cholinesterase; ELISA, enzyme‐linked immunosorbent assay; HE, hematoxylin‐eosin; MDM2, Mouse Double Minute 2 protein; RT‐qPCR, reverse‐transcription quantitative polymerase chain reaction; TBiL, total bilirubin; TUNEL, transferase‐mediated deoxyuridine triphosphate‐biotin nick end labeling.

To better understand the influence of MDM2 in the liver failure process, we first detected the expression changes of MDM2 in the liver tissues of ALF mice by RT‐qPCR and western blot analysis, and it suggested that MDM2 was high‐expressed (Figure 4A,B).

We then injected Ad‐sh‐MDM2 and its NC into ALF mice to suppress MDM2 expression. RT‐qPCR and Western blot analysis uncovered that the injection of Ad‐sh‐MDM2 obviously repressed MDM2 expression in vivo (Figure 4C,D). In response to the silenced MDM2, the level of ALT, AST, and TBiL was depleted, while CHE content in ALF mice was increased (Figure 4E–G). Reduced TNF‐α, IL‐1β, and IL‐6 levels were also observed in serum of ALF mice with silenced MDM2 (Figure 4H). Furthermore, the hepatic cord was arranged radially in the liver tissues of ALF mice, with mild edema of liver cells and clear hepatic sinusoids after downregulating MDM2 (Figure 4I). The cell apoptosis was relieved in ALF mice after MDM2 decrement (Figure 4J).

To sum up, MDM2 was robustly expressed in the liver tissues of ALF mice, and MDM2 silencing contributed to attenuating liver injury and inflammatory response, and blocking the apoptosis of hepatocytes.

### 3.5 MDM2 overexpression reverses the inhibitory effect of miR‐450b‐5p elevation on ALF

**Figure 5:**
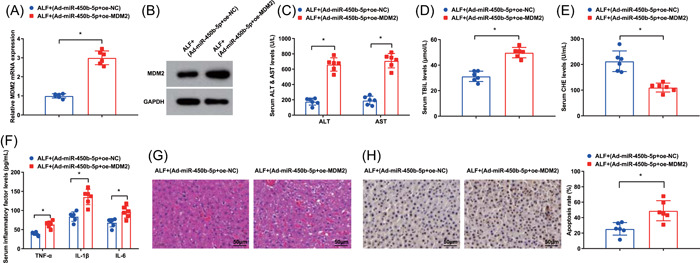
MDM2 overexpression reverses the inhibitory effect of miR‐450b‐5p elevation on liver failure. (A, B) MDM2 expression in ALF mice after injection of Ad‐miR‐450b‐5p + oe‐MDM2 was detected by RT‐qPCR and western blot analysis; (C–E) the concentration of ALT, AST, TBiL, and CHE in ALF mice after injection of Ad‐miR‐450b‐5p + oe‐MDM2 was detected by automatic biochemical analyzer; (F) the contents of serum inflammatory factors in ALF mice after injection of Ad‐miR‐450b‐5p + oe‐MDM2 were examined by ELISA; (G) the pathological changes of liver tissues in ALF mice after injection of Ad‐miR‐450b‐5p + oe‐MDM2 was assessed by HE staining; (H) the hepatocyte apoptosis in ALF mice after injection of Ad‐miR‐450b‐5p + oe‐MDM2 was tested by TUNEL staining. *p < .05. ALF, acute liver failure; ALT, alanine aminotransferase; AST, aspartate aminotransferase; CHE, cholinesterase; ELISA, enzyme‐linked immunosorbent assay; HE, hematoxylin‐eosin; MDM2, Mouse Double Minute 2 protein; RT‐qPCR, reverse‐transcription quantitative polymerase chain reaction; TBiL, total bilirubin; TUNEL, transferase‐mediated deoxyuridine triphosphate‐biotin nick end labeling.

To unravel the relation between MDM2 and miR‐450b‐5p, we probed the impacts of MDM2 overexpression on ALF mice treated with Ad‐miR‐450b‐5p. MDM2 overexpression abrogated the impacts of upregulated miR‐450b‐5p on repressing MDM2 expression as reflected by RT‐qPCR and Western blot analysis (Figure 5A,B). Moreover, MDM2 overexpression also reversed the inhibitory efficacy of elevated miR‐450b‐5p on ALF development (Figure 5C–H).

These outcomes unearthed that MDM2 amplification deteriorated ALF and could abrogate the repressive effects of miR‐450b‐5p elevation on ALF.

## 4 DISCUSSION

ALF is a rare but life‐threatening critical illness that afflicts patients without a liver disease history.^21^ As previously described, there are diverse mechanisms for liver damage‐related disease.^22^, ^23^, ^24^ Anchored in the regulatory mechanism of miR‐450b‐5p in ALF, this research unraveled that upregulated miR‐450b‐5p could repress ALF progression via modulating MDM2.

Initially, we detected miR‐450b‐5p expression in ALF mice, and it was unveiled that miR‐450b‐5p was silenced in ALF mice in comparison to normal mice. Previous studies for directly probing miR‐450b‐5p level in ALF were extremely rare. However, in relapsing‐remitting multiple sclerosis (RRMS), miR‐450b‐5p is also validated to be deficient in the serum of RRMS patients.^25^ In addition, in middle cerebral artery occlusion rats after the transient ischemic attack, miR‐450b‐5p with a depleted expression level has also been observed in cerebrospinal fluid and plasma samples.^12^ Thereafter, we upregulated miR‐450b‐5p to exert its impacts on ALF progression, and it was suggested that miR‐450b‐5p amplification could block ALD development via constraining liver injury degree, mitigating pathological changes, reducing the concentrations of TNF‐α, IL‐1β, and IL‐6 in serum, and decelerating cell apoptosis. Partly in line with our findings, Li et al. have elucidated that, in lung squamous cell carcinoma, miR‐450b‐5p induction contributes to eliciting anticarcinogenic effects via repressing the proliferation, migration, and invasion, yet facilitating the apoptosis of lung squamous cell carcinoma cells.^26^ The inhibitory function of upregulated miR‐450b‐5p in lung adenocarcinoma cell growth has also been evidenced by Zhang et al.^27^ Furthermore, in hepatocellular carcinoma, it has been elucidated that miR‐450b‐5p elevation can promote the apoptosis of hepatocellular carcinoma cells.^28^ Additionally, Lin et al.^29^ have even explicated that miR‐450b‐5p overexpression is effective in ameliorating lung fibrogenesis.

Thereafter, it was predicted that miR‐450b‐5p targeted MDM2 through the bioinformatics website. MDM2 was validated to be highly expressed in ALF mice, and it was interestingly discovered that MDM2 depletion contributed to inhibiting ALF development via mitigating liver injury degree, relieving pathological changes, decreasing the contents of TNF‐α, IL‐1β, and IL‐6 in serum, and repressing cell apoptosis. Limited researchers have ever studied MDM2 function in ALF. However, in liver‐related diseases like hepatocellular carcinoma, MDM2 overactivation has been reported to be associated with hepatocellular carcinoma occurrence.^30^ The robust expression of MDM2 has also been detected in renal parenchymal cells during acute kidney liver.^31^ Functionally, inhibited the ubiquitinylating activity of MDM2 against p53 can block macrophage activation in a murine model of hepatic warm hepatic ischemia‐reperfusion injury.^32^ Additionally, it has been illustrated that MDM2 expression deficiency can protect against hepatocellular carcinoma development via dampening the antioxidant response and apoptosis.^33^ Furthermore, MDM2 blockade also exerts the potential inhibitory influence on wound healing, and tissue repair upon toxic or ischemic injury.^34^ As for the effects of MDM2 on inflammatory response, Wiley et al.^35^ have manifested that MDM2 depletion can reduce the contents of inflammatory cytokines IL‐6 and IL‐1α that are generated by senescent cells, thus dampening cancer progression.

**Figure 6:**
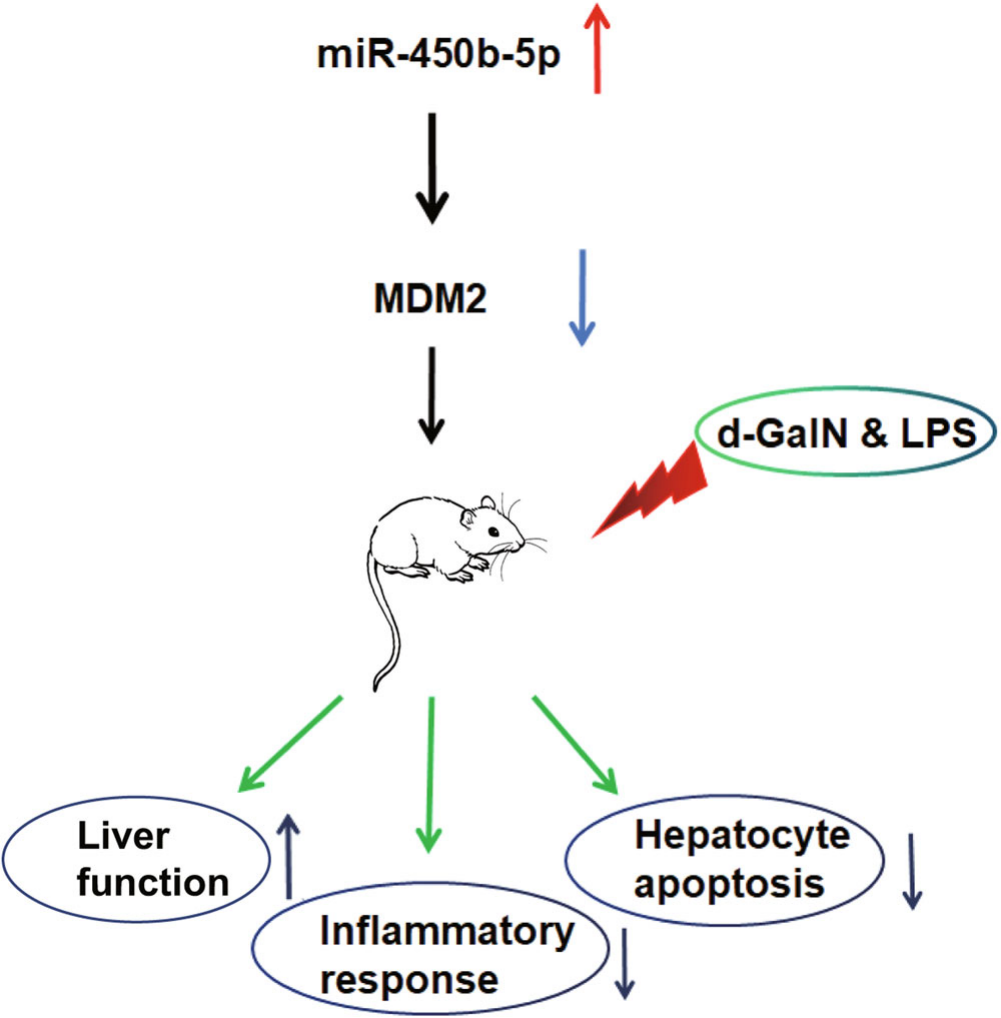
The upregulated miR‐450b‐5p blocks the progression of ALF via targeting MDM2. ALF, acute liver failure; MDM2, Mouse Double Minute 2 protein; LPS, lipopolysaccharide.

In conclusion, this study manifests that miR‐450b‐5p levels are reduced while MDM2 expression is augmented in ALF mice. The augmentation of miR‐450b‐5p can decelerate ALF progression via targeting MDM2 (Figure 6). By highlighting the regulatory mechanism of the miR‐450b‐5p/MDM2 axis in ALF, the current study advances the understanding of the therapeutic strategies of ALF, thereby affording novel research directions for the clinical treatment of ALF. However, there were also limitations in our research. First of all, we did not evaluate the miR‐450b‐5p and MDM2 expression in the clinic, and the exact mechanism in the clinic needs to be further confirmed. Second, one miRNA can target multiple genes, and other potential mechanisms of miR‐450b‐5p in ALF are unknown. Finally, the integrated mechanism of action of the miR‐450b‐5p/MDM2 requires further experimental exploration.

## AUTHOR CONTRIBUTIONS

Jing Kuang contributed to study design; Jun Fang and Shuli Hu contributed to manuscript editing; Xiuhong Yang contributed to experimental studies; Weibo Wan and Jing Li contributed to data analysis. All authors read and approved the final manuscript.

## CONFLICT OF INTEREST STATEMENT

The authors declare no conflict of interest.

## ETHICS STATEMENT

Animal experiments were conducted under the Guide to the Management and Use of Laboratory Animals issued by the National Institutes of Health. The protocol of animal experiments was ratified by the Institutional Animal Care and Use Committee of Wuhan No. 1 Hospital.

## Supporting information

Supporting information.

The targeted binding sequences of miR‐450b‐5p and MDM2 in humans.

## Data Availability

The data that support the findings of this study are available from the corresponding author upon reasonable request.
