# Supplementary material for: Upregulated microRNA‐450b‐5p represses the development of acute liver failure via modulation of liver function, inflammatory response, and hepatocyte apoptosis
Source: Immun Inflamm Dis. 2023 Feb 24;11(2):e767. doi: 10.1002/iid3.767 (PMC9950875; doi:10.1002/iid3.767)
Supplement: Supplementary file 1 — Supporting information. [file IID3-11-e767-s002.doc]

**Supplementary Table 1** The primer sequences used for q-PCR

| Gene | Primer sequence (5’–3’) |
| --- | --- |
| miR-450b-5p | F: 5'- TTTTGCAATATGTTCCTGAATA-3' |
| MDM2 | F: 5′‐CCCGTGAAGGGTCGGAAGAT‐3′ |
|  | R: 5′‐GGTTTTGGTCTAACCTGGAGGC‐3′ |
| GAPDH | F: 5′-GGTGGTCTCCTCTGACTTCAACA-3′ |
|  | R: 5′-ACCAGGAAATGAGCTTGACAAAG-3′ |
| U6 | F: 5′-GGAACGATACAGAGAAGATTA-3′ |
|  | R: 5′-GAGGTATTCGCACCAGAGGA-3′ |

Note: F, forward; R, reverse; miR-450b-5p, microRNA-450b-5p; MDM2, Mouse Double Minute 2 protein; GAPDH, glyceraldehyde phosphate dehydrogenase
