# Supplementary figures and images for: Upregulated microRNA‐450b‐5p represses the development of acute liver failure via modulation of liver function, inflammatory response, and hepatocyte apoptosis
Source: Immun Inflamm Dis. 2023 Feb 24;11(2):e767. doi: 10.1002/iid3.767 (PMC9950875; doi:10.1002/iid3.767)

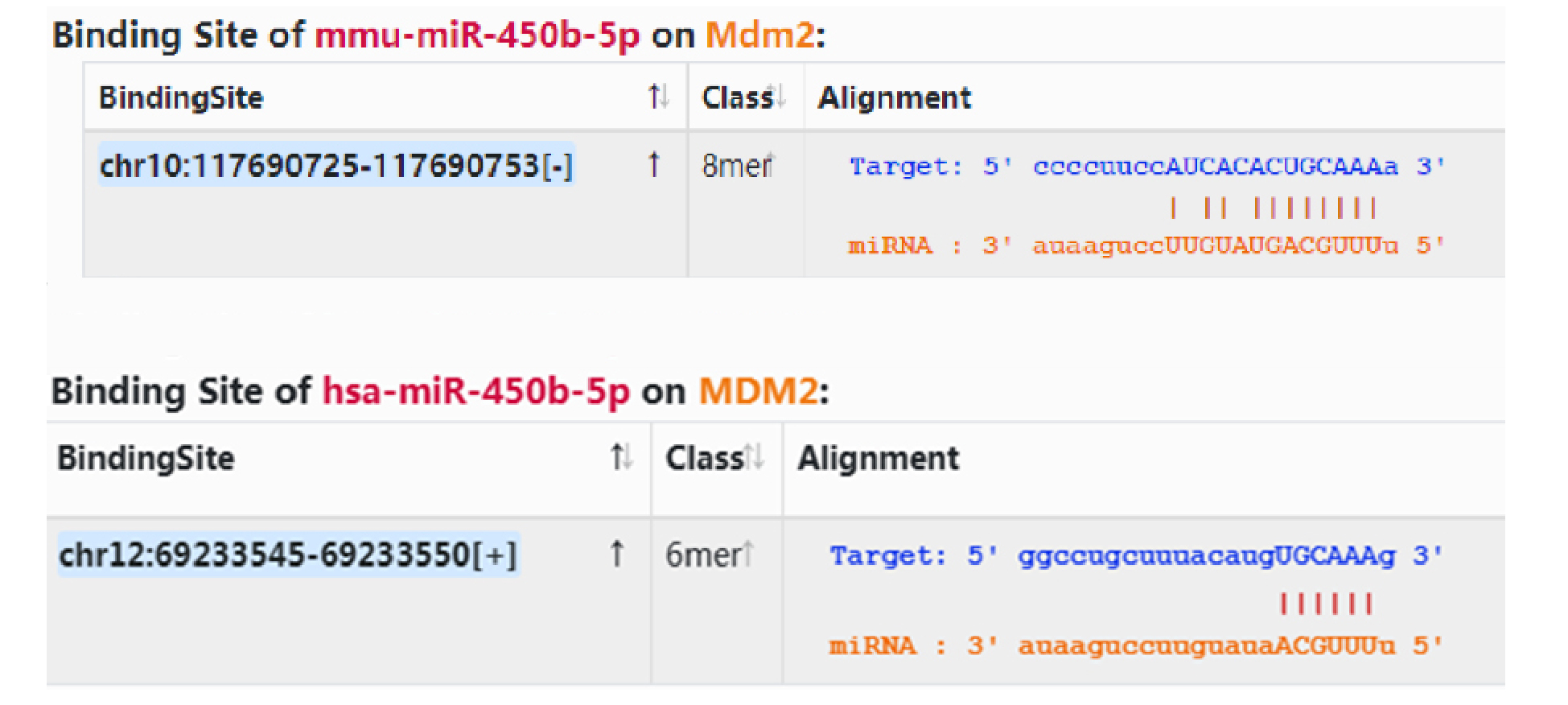

Supplement: Supplementary file 2 — The targeted binding sequences of miR‐450b‐5p and MDM2 in humans. [file IID3-11-e767-s001.jpg]
